# Dental extraction, intensity-modulated radiotherapy of head and neck cancer, and osteoradionecrosis: A systematic review and meta-analysis

**DOI:** 10.1007/s00066-021-01896-w

**Published:** 2022-01-14

**Authors:** Panagiotis Balermpas, Janita E. van Timmeren, David J. Knierim, Matthias Guckenberger, Ilja F. Ciernik

**Affiliations:** 1 Department of Radiation Oncology, Zurich University Hospital, Zurich, Switzerland; 2 Medical School, University of Zürich, Zürich, Switzerland; 3 Center of Dental Medicine, University of Zürich, Zürich, Switzerland; 4 Department of Radiotherapy and Radiation Oncology, Dessau Medical Center, Brandenburg Medical School Theodor Fontane, Dessau, Germany

**Keywords:** Dental care, Osteoradionecrosis prevention, Radiation toxicity, Oropharyngeal cancer, Dental management

## Abstract

**Objective:**

To seek evidence for osteoradionecrosis (ORN) after dental extractions before or after intensity-modulated radiotherapy (IMRT) for head and neck cancer (HNC).

**Methods:**

Medline/PubMed, Embase, and Cochrane Library were searched from 2000 until 2020. Articles on HNC patients treated with IMRT and dental extractions were analyzed by two independent reviewers. The risk ratios (RR) and odds ratios (OR) for ORN related to extractions were calculated using Fisher’s exact test. A one-sample proportion test was used to assess the proportion of pre- versus post-IMRT extractions. Forest plots were used for the pooled RR and OR using a random-effects model.

**Results:**

Seven of 630 publications with 875 patients were eligible. A total of 437 (49.9%) patients were treated with extractions before and 92 (10.5%) after IMRT. 28 (3.2%) suffered from ORN after IMRT. ORN was associated with extractions in 15 (53.6%) patients, eight related to extractions prior to and seven cases related to extractions after IMRT. The risk and odds for ORN favored pre-IMRT extractions (RR = 0.18, 95% CI: 0.04–0.74, *p* = 0.031, I^2^ = 0%, OR = 0.16, 95% CI: 0.03–0.99, *p* = 0.049, I^2^ = 0%). However, the prediction interval of the expected range of 95% of true effects included 1 for RR and OR.

**Conclusion:**

Tooth extraction before IMRT is more common than after IMRT, but dental extractions before compared to extractions after IMRT have not been proven to reduce the incidence of ORN. Extractions of teeth before IMRT have to be balanced with any potential delay in initiating cancer therapy.

**Supplementary Information:**

The online version of this article (10.1007/s00066-021-01896-w) contains supplementary material, which is available to authorized users.

## Introduction and objectives

Ionizing radiation (IR) inhibits wound healing and damaged irradiated tissue has reduced healing abilities [1]. IR of the oral and pharyngeal mucosa and the salivary glands leads to changes of the oral milieu, including decreasing pH and saliva quantity, and changes in bacterial composition [2, 3]. Oral hygiene is helpful for preserving teeth and reduces adverse side effects of radiotherapy (RT) [4]. A severe side effect is osteoradionecrosis (ORN), characterized by the exposure and devitalization of the bone, causing severe pain, swelling, or difficulties with eating. Poor dental status predisposes for dental decay and ORN if left untreated during, prior to, or after RT [5–7]. Therefore, it has become routine to evaluate the dental status and to extract non-restorable teeth prior to any high-dose radiotherapy for head and neck cancer (HNC) [4].

The rationale for extracting critical teeth before RT is that bone heals better before irradiation than after irradiation. In earlier studies, tooth extractions after RT have been reported to be associated with a high risk of ORN [8]. However, some authors observed that dental extractions prior to RT do not prevent ORN completely [9–11]. Currently, there is no conclusive evidence that dental extractions before irradiation offer a significant risk reduction for the development of ORN compared to dental extractions after RT, and there are no randomized controlled trials [12]. Reviews hold back on meta-analyses, and patients treated in the pre-intensity-modulated radiotherapy (IMRT) era were included [4, 13].

Nabil and Samman reported that the incidence of ORN varied between 12.9% in 1938 to almost 40% in the 1960s, and fell to 8.2% in 2003 [14]. More recent studies reported an ORN incidences of 0–5% after the introduction of intensity-modulated radiotherapy (IMRT) [15–17]. As IMRT represents a modern RT technique that can balance the dose of IR within small spatial volumes, it is unclear how technological advancements in the last decades have affected the need or the sequencing of dental care in the context of IMRT [18]. In this systematic review and meta-analysis using published patient data, we reviewed the existing literature in order to answer the question of whether dental extractions prior to intensity-modulated radiotherapy effectively decrease the risk of developing ORN.

## Materials and methods

### Data and data sources

This systematic review was conducted according to the PRISMA (Preferred Reporting Items for Systematic Reviews and Meta-Analyses) guideline [19]. In September 2019, a first search was conducted for the Medline/PubMed, the Embase, and the Cochrane Library online databases for articles published between 2000 and 2020 which report on head and neck cancer patients with dental extractions undergoing IMRT and developing ORN afterwards. The search included the specific arrangement of free terms and medical subject headings (MeSH) terms in the population, intervention, control, and outcome search design (PICO). Details of the search sequence are given in the supplementary figure Appendix 1.

### Selection of studies

IMRT was introduced in the late 1990s. All published articles from the year 2000 onwards were included. Previous IMRT-only studies revealed an ORN incidence of approximately 5% [15–17]. Therefore, case reports and studies with less than 20 IMRT patients were excluded. Exact eligibility criteria are also listed under “Supplementary methods.”

## Results

**Fig. 1:**
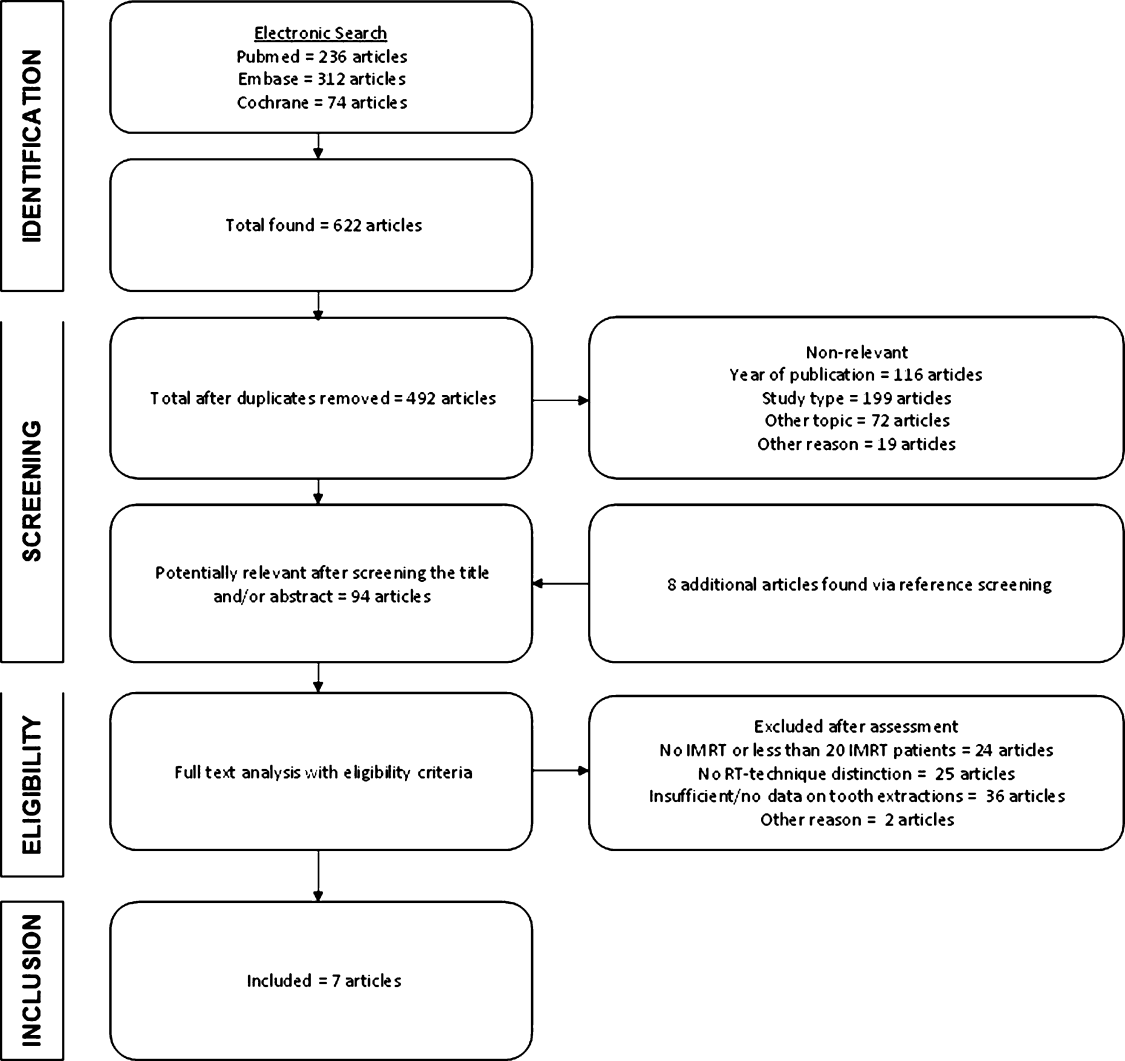
Preferred Reporting Items for Systematic Reviews and Meta-Analyses (PRISMA) flowchart. *IMRT* intensity-modulated radiotherapy, *RT* radiotherapy

A first search was conducted on 28 September 2019 and a second search on 21 January 2020. As shown in the PRISMA flowchart (Fig. 1), 236 articles were found via Medline/PubMed, 312 articles via Embase, and 74 articles via the Cochrane library resulting in a total of 622 articles. After deleting duplicates, 492 articles remained for further processing. The articles were checked for the year of publication, the study type, and the main topic in the title and the abstract. Finally, 86 articles were submitted to qualitative analysis of the text. Eight additional articles were found in the references, resulting in 94 articles.

After analysis and correspondence with authors, eligibility criteria were applied and 87 articles were excluded: 24 articles included patients without IMRT or IMRT was used in less than 20 patients; in 25 studies, distinguishing between patients treated with IMRT or other techniques was not possible for the entire cohort, only for specific subgroups, and absolute figures regarding ORN cases and extractions for IMRT were impossible; in 36 articles, there were incomplete data on dental extractions and individual ORN; the remaining 2 studies marked “other reasons” only reported on ORN grade ≥ 3 [10]. After the eligibility assessment, 7 studies that met our eligibility criteria were suitable for inclusion (Table 1).

**Table 1:** Literature articles included

Author	Year	Title	Study design	Location	Period
Ben-David et al. [19]	2007	Lack of osteoradionecrosis of the mandible after intensity-modulated radiotherapy for head and neck cancer: likely contributions of both dental care and improved dose distributions	Retrospective Single center	USA Michigan Ann Arbor	1996–2005
Gomez et al. [20]	2011	Correlation of osteoradionecrosis and dental events with dosimetric parameters in intensity-modulated radiation therapy for head and neck cancer	Retrospective Single center	USA New York	2000-2007
Maesschalck et al. [21]	2016	Comparison of the incidence of osteoradionecrosis with conventional radiotherapy and intensity-modulated radiotherapy	Retrospective Single center	Switzerland Geneva	2002–2012
Muraki et al. [22]	2019	Dental intervention against osteoradionecrosis of the jaws in irradiated patients with head and neck malignancy; a single-arm prospective study	Prospective Single center	Japan Kobe	2015–2016
Schuurhuis et al. [23]	2018	Patients with advanced periodontal disease before intensity-modulated radiation therapy are prone to develop bone healing problems; a 2-year prospective follow-up study	Prospective Single center	Netherlands Groningen	2011–2013
See Toh et al. [24]	2018	Dental extractions for preradiation dental clearance and incidence of osteoradionecrosis in patients with nasopharyngeal carcinoma treated with intensity-modulated radiotherapy	Retrospective Single center	Singapore Singapore	2011–2013
Willaert et al. [25]	2019	Does intensity-modulated radiation therapy lower the risk of osteoradionecrosis of the jaw? A long-term comparative analysis	Retrospective Single center	Belgium Leuven	2003–2010

The percentage of patients who had to undergo dental extractions before irradiation ranged from 18% [17, 26] to 90% [27] of the respective study populations. Post-IMRT extractions ranged from 7% [17, 27] to 22% [28]. Combined, 875 patients were treated, with 432 of them having dental extractions before irradiation versus 92 afterwards (Table 2).

**Table 2:** Dental extractions before and after intensity-modulated radiotherapy (IMRT)

Author	Total IMRT patients, *n*	Edentulous at presentation, *n* (%)	Patients with extractions prior to IMRT, *n* (%)	Patients without extractions prior to IMRT, *n* (%)	Patients with extractions after IMRT, *n* (%)	Patient without extractions after IMRT, *n* (%)^d^
Ben-David et al. [19]^a^	176	16 (9)	31 (18)	122 (69)	13 (7)	163 (93)
Gomez et al. [20]	168	7 (4)	30 (18)	138 (82)	20 (12)	148 (88)
Maesschalck et al. [21]^a^	89	20 (22)	46 (52)	39 (44)	9 (10)	80 (90)
Muraki et al. [22]	46	0	24 (52)	22 (48)	4 (9)	42 (91)
Schuurhuis et al. [23]	56	0	43 (77)	13 (23)	6 (11)	50 (89)
See Toh et al. [24]^b^	231	0	207 (90)	25 (11)	16 (7)	215 (93)
Willaert et al. [25]^c^	109	16 (15)	51 (47)	40 (37)	24 (22)	85 (78)
Total	875	59 (7)	432 (49)	394 (45)	92 (11)	783 (89)

*IMRT* intensity-modulated radiotherapy

^a^The preradiation dental extraction percentages do not add up to 100% because extraction data were not available/known for some patients

^b^Reported combined more cases as the total population

^c^The preradiation dental extraction percentages do not add up to 100% because edentulous patients were not counted as “Patients without extractions prior to IMRT” and dental status was not available for 1 patient

^d^Calculated (total IMRT patients–patients with extractions after IMRT)

Overall, 28 (3.2%) patients developed osteoradionecrosis. Based on the authors’ statements or the localization of the ORN, eight ORN cases can be attributed to extractions prior to IMRT. Seven ORN cases can be accounted for by post-IMRT extractions. The remaining 13 cases were not triggered by dental extraction according to the reports (Table 3).

**Table 3:** Timeline of dental extractions and manifestation of osteoradionecrosis (ORN)

Author	Primary tumor location	TN stage	ORN location	ORN onset after IMRT (months)	Patient had pre-IMRT extraction	Patient had post-IMRT extraction	Pre-IMRT extraction related to ORN	Post-IMRT extraction related to ORN
Ben-David et al. [19]	No ORN observed							
Gomez et al. [20]	Floor of mouth	T2N1	Mandible	31	No	n.a.	No	n.a.
	Floor of mouth	T2N2b	Mandible	32	No	n.a.	No	n.a.
Maesschalck et al. [21]	Oropharynx	T1 (*n* = 0) T2 (*n* = 2) T3 (*n* = 1) T4 (*n* = 6)	Mandible	54	n.a.	Yes	No	Yes
	Oropharynx		Mandible	23	n.a.	No	No	No
	Oropharynx		Mandible	7	n.a.	Yes	No	Yes
	Oropharynx		Mandible	n.a.	n.a.	Yes	No	No
	Oropharynx		Mandible	32	Probably	No	n.a.	No
	Oropharynx		Mandible	8	n.a.	Yes	No	Yes
	Oropharynx		Mandible	28	No	No	No	No
	Oropharynx		Mandible	31	n.a.	Yes	No	Yes
	Oropharynx		Mandible	2	Yes	No	Yes	No
Muraki et al. [22]	Hypopharynx	T3N0	Mandible	3	No	No	No	No
	Oropharynx	T4aN2b	Mandible	11	No	No	No	No
	Oropharynx	T3N0	n.a.	18	Yes	Yes	No	No
Schuurhuis et al. [23]	Oral Cavity or Oropharynx	n.a.	Mandible	3	Yes	n.a.	Yes	No
		n.a.	Mandible	7	n.a.	Yes	No	Yes
		n.a.	Mandible	2	n.a.	n.a.	No	No
		n.a.	Transplant	2	n.a.	n.a.	No	No
See Toh et al. [24]	Nasopharynx	T1 (*n* = 1) T2 (*n* = 0) T3 (*n* = 2) T4 (*n* = 3)	Maxilla	10	Yes	No	5/6 Yes ^a^	No
	Nasopharynx		Mandible	6	Yes	No		No
	Nasopharynx		Mandible	3	Yes	No		No
	Nasopharynx		Mandible	1	Yes	No		No
	Nasopharynx		Mandible	24	Yes	No		No
	Nasopharynx		Mandible	4	Yes	No		No
Willaert et al. [25]	Oropharynx (*n* = 3) Hypopharynx (*n* = 1)	n.a.	Mandible	51.6	n.a.	Yes	No	Yes
		n.a.	Mandible	54.3	Yes	n.a.	Yes	No
		n.a.	Mandible	67.7	n.a.	n.a.	No	No
		n.a.	Mandible	6.7	n.a.	Yes	No	Yes

*IMRT* intensity-modulated radiotherapy, *n.a*. not available

^a^ In five of six cases, patients had teeth removed prior to irradiation at the areas that subsequently developed ORN. One case occurred spontaneously

Ben-David et al. [17] reported that dental records for 174 out of 176 patients were analyzed. While 16 patients presented edentulous, 157 were dentulous during IMRT. Dental extractions prior to IMRT were received by 30 patients, with two being the median number of teeth removed (range 1–8); 122 patients started IMRT without prior extractions; and 13 patients had extractions after irradiation. With a median follow-up of 34 months, they had no case of grade 2 or worse ORN (CTCAE v3.0) in the 176 patients. They did not observe any grade 1 ORN, because post-treatment panoramic X‑rays were taken only on clinical suspicion of ORN and not systematically.

Gomez et al. reported data for 168 patients [26]: 30 patients received extractions prior to IMRT and 20 patients after IMRT. Seven patients presented edentulous and 138 patients started IMRT without extractions. With a median follow-up of 37.4 months, they observed two cases of ORN, 31 and 32 months after the end of IMRT. None of the patients with ORN had pre-IMRT extractions and no patient underwent bone stripping as part of the surgical procedure.

Maesschalck et al. analyzed dental extraction reports available for 85 of 89 patients [29]: 46 of them received pre-IMRT extractions, nine patients received extraction after irradiation, and 20 patients were edentulous before IMRT. With a median follow-up of 3.2 (± 1.8) years, they observed nine cases of ORN in the 89 patients. ORN occurred with a median of 25 months (range 8–54 months). The authors stated on request that one patient had a dental extraction prior to IMRT which did not heal well, leading to ORN 2 months after IMRT. In four patients, dental extractions after IMRT were the trigger for ORN. In the remaining four cases, no connection between extraction and ORN was observed.

Muraki et al. specified data after request. Prior to IMRT, 24 out of 46 patients underwent extractions [30]. Four patients received post-IMRT extractions. In total, 97 teeth were extracted in the IMRT group. On completion of the study after 2 years, they observed three cases of jawbone exposure. Two patients had neither dental extraction before nor after IMRT. The first case of ORN occurred 3 months after IMRT in a patient with a lingual anterior lesion of the mandible; however, this was unrelated to the dentition status. The bone exposure healed within a month after a sequestrectomy was performed. The second case was due to an impacted wisdom tooth, not extracted prophylactically, and led to bone exposure 11 months after IMRT. This exposure healed after surgical debridement over a period of 13 months. The third patient had pre- and post-IMRT extractions and 18 months after IMRT, ORN was observed. Surgical trauma or dental extractions could not be causally linked ORN. Bone resorption led to a floating tooth, which was lost spontaneously, and bone exposure probably existed before the loss. The treatment from diagnosis of ORN until healing took 5 months (Table 3).

Schuurhuis et al. reported that of 56 patients, 43 were submitted to pre-IMRT extractions [20]. Five had full mouth clearance, rendering them edentulous. Median number of teeth extracted was seven (range 2–10 teeth). Six patients had one to three teeth extracted after IMRT. On completion of the study, with a median follow-up of 24 months (range 11–27 months), 10 patients with bone healing problems were reported. Three patients were diagnosed with delayed wound healing after pre-IMRT dental extraction, three with lingual mandibular sequestration (unrelated to dental extraction), and four with ORN. The first case of ORN occurred 3 months after IMRT at the site where a mandibular molar was extracted prior to irradiation. The second case developed 7 months after IMRT because of a non-healing socket, which was the result of a post-IMRT extraction. Another patient had pathological fracture 2 months after IMRT, where idiopathic ORN had preceded. The fourth case of ORN occurred in the transplanted fibula and thus was not related to pre-or post-IMRT dental extractions.

See Toh et al. reported on 231 patients, 207 needing extractions prior to IMRT, and 16 patients receiving post-IMRT extractions [27]. A total of 943 teeth were extracted, 4.1 teeth extracted per patient on average. With an average follow-up duration of 52 months, they observed six cases of ORN. Median duration for ORN to occur was 5 months (range 1–24 months). The authors stated that 5 patients had pre-IMRT extractions at the sites that developed ORN and that ORN was possibly the result of insufficient healing time of the extraction wounds before the start of IMRT.

Willaert et al. reported on 108 patients with documented details on their dental status out of a cohort of 109 patients treated with IMRT [28]. While 16 patients presented edentulous, 51 of 92 dentulous patients were treated with extractions prior to IMRT and 40 patients started irradiation without dental extractions; 24 patients underwent post-IMRT extractions. With a mean follow-up of 44.4 months (range 6–96 months), 4 patients suffered from ORN. Median interval to ORN diagnosis was 40 months (range 6.7–67.7 months). The authors stated that three out of four cases were related to dental extractions. One ORN case occurring after 54.3 months was related to extraction before, and two cases, occurring after 6.7 and 51.6 months, were related to extraction after IMRT.

### Risk of developing dental extraction-related osteoradionecrosis

**Fig. 2:**
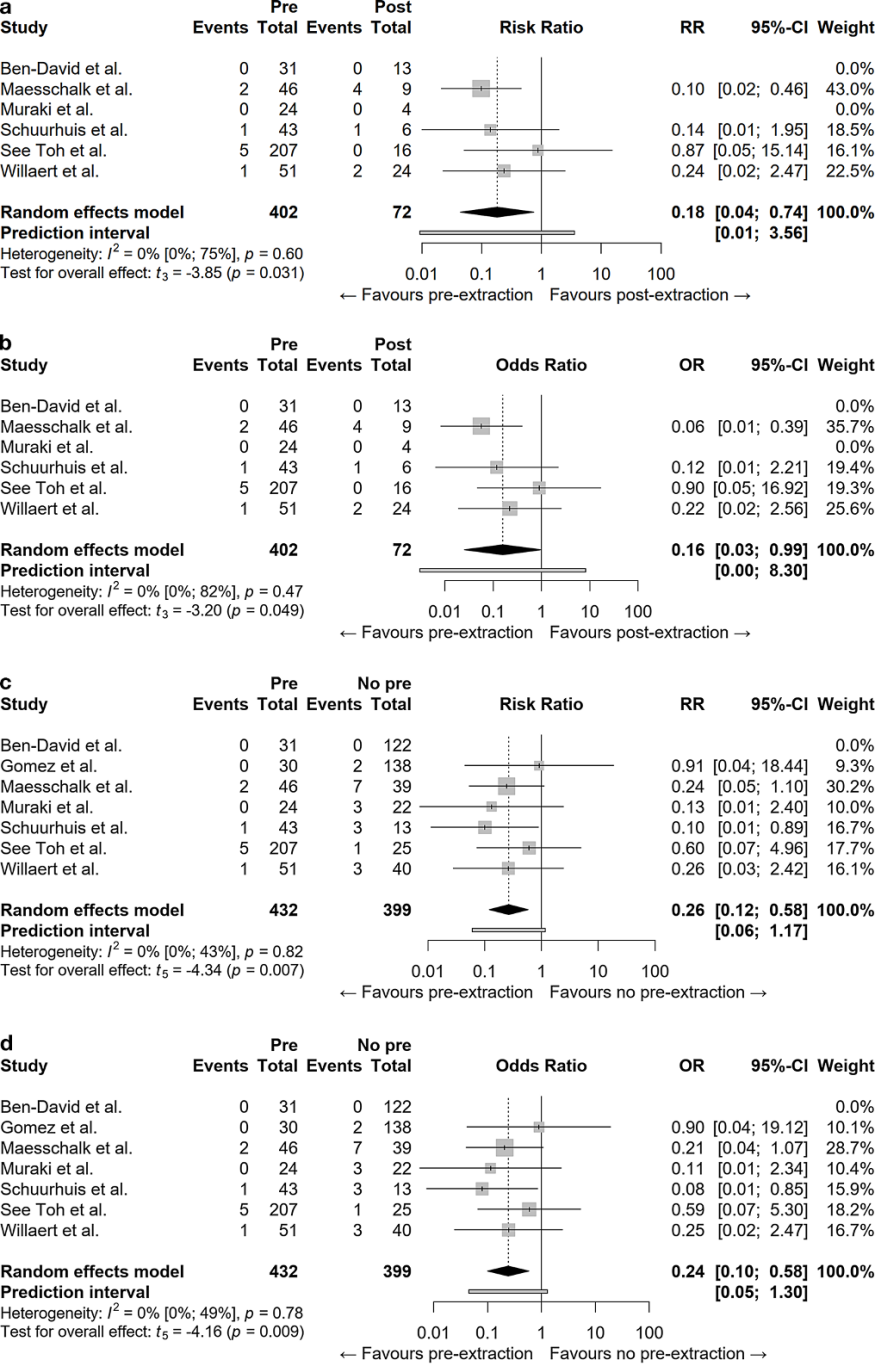
**a** Forest plot for the risk ratio (*RR*) of ORN development between pre-intensity-modulated radiotherapy (*IMRT*) extractions and post-IMRT extractions. **b** Forest plot for the odds ratio (*OR*) of ORN development between pre-IMRT extractions and post-IMRT extractions. **c** Forest plot for the RR of ORN development between pre-IMRT extractions and no pre-IMRT extractions. **d** Forest plot for the OR of ORN development between pre-IMRT extractions and no pre-IMRT extractions. *CI* confidence interval

Significantly more (*p* < 0.001) patients underwent extraction before (*n* = 432) than after IMRT (*n* = 92). Further, having extractions prior to IMRT was associated with ORN development less often (8/432) than having extractions after IMRT (7/92) (*p* < 0.01). As shown in Fig. 2, the pooled risk ratio and odds ratio for ORN development after pre-IMRT extractions to ORN development after post-IMRT extractions were 0.18 (*p* = 0.031) and 0.16 (*p* = 0.049), respectively.

Having pre-IMRT extractions resulted less often in ORN than not having pre-IMRT extractions. By taking all ORN cases into account which were not triggered by pre-IMRT extraction and comparing the patients who did not have pre-IMRT extractions (“No pre” in Fig. 2) to the pre-IMRT extraction group, the risk ratio and odds ratio also indicate an advantage for pre-IMRT extractions (RR = 0.26, *p* = 0.007, OR = 0.24, *p* = 0.009).

Lastly, as the prediction interval for the risk ratio and odds ratio includes the value one, no difference in the risk and odds of ORN-development between having extractions prior to IMRT, compared to having extractions after IMRT may be present upon repetition of testing. The same applies to having extractions prior to IMRT, compared to not having extractions prior to IMRT.

## Discussion

Radiation-related damage to dentition is of multifactorial origin [21]. Adequate oral hygiene helps to prevent tooth decay and subsequent threats to the bone underneath [22, 23]. Oral hygiene impacts on the risk of caries, and thus on ORN [26]. Dental extractions can enhance ORN and dental extraction prior to IMRT seems to come with a reduced risk compared to dental extraction after IMRT in the present review of the literature. Our observation is in line with the prevailing opinion and current practice, but in contrast to recent reports [9–11]. The statistical difference in OR and RR was not large enough to be conclusive. All articles included in the present meta-analysis report on dental evaluation to identify critical teeth and had hygiene protocols during and after irradiation to prevent dental sequelae. Attribute differences in numbers of patients undergoing pre-IMRT extractions and post-IMRT extractions were present. A general consensus to extract critical teeth before radiotherapy on the one hand, and short follow-up duration and patients changing the treating institution after irradiation on the other hand being the most common of these. Thus, we cannot exclude that a detrimental effect of dental extractions prior to IMRT may be compensated by improved radiotherapy-techniques. The current data suggest that it seems to be premature to conclude that dental extractions before IMRT reduce the risk for ORN development after IMRT. Finally, the protocols from the analyzed studies regarding the pre-irradiation dental extractions and the introduction of the IMRT-technique result in an incidence of less than 5% of ORN, which leaves little room for improvement (Table 4**; **Refs. [5, 15–17, 20, 24–40]).

**Table 4:** Osteoradionecrosis (ORN) incidence in studies with intensity-modulated radiotherapy (IMRT) patients

Author	Year	IMRT patients (*n*)	ORN cases (*n*)	Percentage (%)
Ben-David et al. [19]	2007	176	0	0.0
Huang et al. [31]	2008	71	1	1.4
Eisbruch et al. [32]	2009	69	3	4.3
Mendelhall et al. [41]	2010	130	4	3.1
Montejo et al. [33]	2010	43	1	2.3
Gomez et al. [20]	2011	168	2	1.2
Studer et al. [18]	2011	304	5	1.6
Nguyen et al. [34]	2012	83	1	1.2
Tsai et al. [35]	2012	334	21	6.3
Chen et al. [36]	2015	1692	105	6.2
Maesschalk et al. [21]	2016	89	9	10.1
Monroe et al. [37]	2016	89	4	4.5
Owosho et al. [26]	2016	1023	44	4.3
Caparrotti et al. [38]	2017	1196	71	5.9
Kojima et al. [8]	2017	26	1	3.8
Mohamed et al. [39]	2017	1700	83	4.9
Moon et al. [40]	2017	225	9	4.0
Schuurhuis et al. [23]	2017	56	4	7.1
See Toh et al. [24]	2017	231	6	2.6
Zhang et al. [42]	2017	534	41	7.7
Muraki et al. [22]	2019	46	3	6.5
Willaert et al. [25]	2019	109	4	3.7
Total	–	8394	422	5.0

The current low rates of ORN since the introduction of IMRT put the focus on improvement and standardization of methods of detecting and reporting ORN. Similarly, the criteria for dental extraction must also be adapted for IMRT. A study conducted at the University Hospital of Zurich in 2011 compared two protocols for dental extractions in IMRT patients and showed that with the risk-adapted dental care treatment (RaDC), fewer teeth can be extracted (with 50% more patients receiving no extraction at all) without increasing the incidence of ORN [15]. However, the mean and median follow-up in the RaDC group was shorter (19/13 months) compared to the group with the conventional protocol (40/30 months). Therefore, protocols that motivate practitioners to easily extract teeth prior to RT should be discouraged. In times of an epidemiological shift from older and sometimes indifferent head and neck cancer patients with tobacco- and alcohol-induced tumors to younger human papilloma virus(HPV)-induced tumor patients, leaving as many teeth in place as possible may become more important in terms of life quality.

### Limitations of this systematic review

The literature search for this systematic review included articles in English or German only, representing a linguistic selection bias, and several authors were not attenable to our requests for additional information on the data.

There is no suitable tool to assess the risk of bias of included studies and our approach to data interpretation. As five of seven studies were conducted retrospectively, a retrospective bias applies. The data presented in this review reflect the information provided by the authors of the studies. A selection bias for the retrospective selection of the patient population and the inclusion criteria is likely. Furthermore, a detection bias must also be present because ORN was defined and graded differently in various studies. Three patients with ORN out of two studies included in the present meta-analysis had ORN with unknown association with dental extractions. We decided to include the reports and put them in the group, which was the most accurate with the available information.

Moreover, the result is influenced by many confounders that we were unable to integrate into the calculation because of a lack of data. Such known confounders are, amongst others and not limited to, the exact dose at each extraction site, exact time interval between tooth extraction and treatment, nicotine and alcohol consumption, concomitant systemic treatments, and personal oral hygiene status at diagnosis.

## Conclusion

Osteoradionecrosis (ORN) after intensity-modulated radiotherapy (IMRT) has become a rare complication. There is no conclusive evidence that dental extractions after IMRT will result in a higher risk of ORN than dental extractions prior to IMRT. However, a reduced risk of developing ORN with pre-IMRT dental extractions emerges after analysis of the current literature, because all research groups use pre-IMRT dental evaluation. Thus, pre-radiotherapy (RT) dental care and extractions remain the standard procedure to prevent dental complications from IMRT. To prove that pre-IMRT extractions decrease the risk of developing ORN, randomized, clinical trials with sufficient follow-up durations would be necessary, but unlikely to be performed due to the low incidence of ORN. Pre-RT dental extractions must be weighed against postponing cancer treatment and the chances of successful control of cancer on the basis of an interdisciplinary evaluation, setting priorities according to individual risks.

## Supplementary Information

1. Appendix 1: Search terms

2. Supplementary methods
