# Supplementary material for: Dental extraction, intensity-modulated radiotherapy of head and neck cancer, and osteoradionecrosis: A systematic review and meta-analysis
Source: Strahlenther Onkol. 2022 Jan 14;198(3):219–28. doi: 10.1007/s00066-021-01896-w (PMC8863691; doi:10.1007/s00066-021-01896-w)
Supplement: Supplementary file 1 — 1. Appendix 1: Search terms [file 66_2021_1896_MOESM1_ESM.pdf]

Appendix 1: Search terms

Medline/PubMed (Search 1)	Population:	#1	((("head and neck neoplasms/radiotherapy"[MeSH Terms])) OR "cranial irradiation"[MeSH Terms]) OR "radiation therapy"
	Intervention:	#2	((((((((("tooth extraction") OR "teeth extraction") OR "dental extraction") OR "extraction of teeth") OR "exodontia") OR "exodontics") OR "molar extraction") OR "odontoectomy") OR "tooth removal") OR "tooth resection"
	Comparison:		Not applied
	Outcome:		Not applied
	Language:	#3	("german"[Language]) OR "english"[Language]
	Search querie:		#1 AND #2 AND #3
	Search date:		28.09.19, no restriction applied
	Number of articles found (n):		191
Medline/PubMed (Search 2)	Population:	#1	radiotherapy, intensity modulated[MeSH Terms]
	Intervention:	#2	Not applied
	Comparison:	#3	Not applied
	Outcome:	#4	"osteoradionecrosis"[MeSH Terms]
	Search querie:		#2 AND #4
	Search date:		21.01.20, no restriction applied
	Number of articles found (n):		45
Embase (Search 1)	Population:	#1	('skull irradiation'/exp OR 'cranial irradiation' OR 'irradiation, cranial' OR 'skull irradiation' OR 'skull radiation' OR 'radiotherapy'/exp OR 'bioradiant therapy' OR 'bucky irradiation' OR 'bucky radiation' OR 'bucky radiotherapy' OR 'bucky ray' OR 'bucky ray radiation' OR 'bucky therapy' OR 'fractionated radiotherapy' OR 'hemibody irradiation' OR 'hypophysectomy, radiation' OR 'hypophysis irradiation' OR 'hypophysis radiation' OR 'irradiation therapy' OR 'irradiation treatment' OR 'irradiation, hypophysis' OR 'lymphatic irradiation' OR 'pituitary irradiation' OR 'radiation beam centration' OR 'radiation repair' OR 'radiation therapy' OR 'radiation treatment' OR 'radio therapy' OR 'radio treatment' OR 'radiohypophysectomy' OR 'radiology, therapeutic' OR 'radiotherapy' OR 'radiotherapy setup errors' OR 'radiotreatment' OR 'roentgen irradiation, therapeutic' OR 'roentgen therapy' OR 'roentgen treatment' OR 'rontgen therapy' OR 'therapeutic radiology' OR 'therapy, irradiation' OR 'therapy, radiation' OR 'therapy, roentgen' OR 'treatment, irradiation' OR 'treatment, radiation' OR 'treatment, roentgen' OR 'x radiotherapy' OR 'x ray therapy' OR 'x ray treatment' OR 'x-ray therapy')
	Intervention:	#2	('teeth extraction'/exp OR 'tooth extraction'/exp OR 'dental extraction' OR 'dental extractions' OR 'exodontia' OR 'exodontics' OR 'extraction, tooth' OR 'molar amputation' OR 'molar extraction' OR 'odontectomy' OR 'tooth extraction' OR 'tooth removal' OR 'tooth resection')
	Comparison:		Not applied
	Outcome:		Not applied
	Filter:	#3	([adult]/lim OR [aged]/lim OR [middle aged]/lim OR [young adult]/lim) AND ([english]/lim OR [german]/lim) AND [humans]/lim
	Search querie:		#1 AND #2 AND #3
	Search date:		28.09.19, no restriction applied
	Number of articles found (n):		273

Embase (Search 2)	Population:	#1	('intensity modulated radiation therapy'/exp OR 'imrt' OR 'intensity modulated arc therapy' OR 'intensity modulated photon radiotherapy' OR 'intensity modulated radiation therapy' OR 'intensity modulated radiotherapy' OR 'intensity modulated therapy' OR 'intensity-modulated radiation therapy' OR 'intensity-modulated radiotherapy' OR 'radiotherapy, intensity modulated' OR 'radiotherapy, intensity-modulated')
	Intervention:	#2	('tooth extraction'/exp OR 'dental extraction' OR 'dental extractions' OR 'exodontia' OR 'exodontics' OR 'extraction, tooth' OR 'molar amputation' OR 'molar extraction' OR 'odontectomy' OR 'tooth extraction' OR 'tooth removal' OR 'tooth resection')
	Comparison:	#3	Not applied
	Outcome:	#4	('bone necrosis'/exp OR 'bone necrosis' OR 'necrosis, bone' OR 'osteonecrosis' OR 'osteoradionecrosis')
	Search querie:		#1 AND #2 AND #4
	Search date:		21.01.20, no restriction applied
	Number of articles found (n):		39
Cochrane Library	Population:		#1 MeSH descriptor: [Head and Neck Neoplasms] explode all trees and with qualifier(s): [radiotherapy - RT]<br>#2 MeSH descriptor: [Radiotherapy] explode all trees and with qualifier(s): [adverse effects - AE]<br>#3 MeSH descriptor: [Cranial Irradiation] explode all trees and with qualifier(s): [adverse effects - AE]<br>#4 radiation therapy<br>#5 irradiated patients<br>#6 children
	Intervention:		#7 MeSH descriptor: [Tooth Extraction] explode all trees<br>#8 dental extraction<br>#9 exodontia<br>#10 exodonticon<br>#11 molar amputation<br>#12 molar extraction<br>#13 odontectomy<br>#14 tooth removal<br>#15 tooth resection
	Comparison:		Not applied
	Outcome:		Not applied
	Language:		Not applied
	Search querie:		{OR #1-#5} AND {OR #7-#15} NOT #6
	Search date:		28.09.19, no restriction applied
	Number of articles found (n):		74
