# Supplementary material for: Dental extraction, intensity-modulated radiotherapy of head and neck cancer, and osteoradionecrosis: A systematic review and meta-analysis
Source: Strahlenther Onkol. 2022 Jan 14;198(3):219–28. doi: 10.1007/s00066-021-01896-w (PMC8863691; doi:10.1007/s00066-021-01896-w)
Supplement: Supplementary file 2 — 2. Supplementary methods [file 66_2021_1896_MOESM2_ESM.docx]

**Eligibility criteria**

In order to answer our question, studies were required, that recorded dental extractions before and after IMRT for the entire study population. Furthermore, all cases of ORN, which occurred due to pre- or post-IMRT extractions. This way it would be possible to draw a conclusion as to whether more ORN-cases occur in either group. The eligibility criteria have been designed to identify such studies. Following criteria were mandatory for inclusion or led to the exclusion of the articles found in the literature search.

Inclusion criteria:

• The article was published in the year 2000 or later

• The article language was in English or German

• The study dealt with participants undergoing intensity-modulated radiotherapy (IMRT) for head and neck cancer, with any dose exposure for the jaws

• The study included patients, who received dental extractions during the treatment or follow-up period

• The study reported on the incidence of ORN (any grade) in the jaws

• For each patient with ORN, the study reported whether an association between dental extraction and ORN was present or not

• The study provided information on dental extractions pre- or post-IMRT for the whole study population

**Exclusion criteria**

• The study included children only, animal models or in-vitro investigations

• The article was an audit, case report, comment, editorial contribution, letter, review, statement or survey

The study included less than 20 participants
